# Stereo Vision Based Sensory Substitution for the Visually Impaired

**DOI:** 10.3390/s19122771

**Published:** 2019-06-20

**Authors:** Simona Caraiman, Otilia Zvoristeanu, Adrian Burlacu, Paul Herghelegiu

**Affiliations:** Faculty of Automatic Control and Computer Engineering,“Gheorghe Asachi” Technical University of Iasi, D. Mangeron 27, 700050 Iasi, Romania; sarustei@cs.tuiasi.ro (S.C.); otiliajacota@gmail.com (O.Z.); pherghelegiu@tuiasi.ro (P.H.)

**Keywords:** visual impairment, sensory substitution, stereo vision, 3D reconstruction, obstacle detection

## Abstract

The development of computer vision based systems dedicated to help visually impaired people to perceive the environment, to orientate and navigate has been the main research subject of many works in the recent years. A significant ensemble of resources has been employed to support the development of sensory substitution devices (SSDs) and electronic travel aids for the rehabilitation of the visually impaired. The Sound of Vision (SoV) project used a comprehensive approach to develop such an SSD, tackling all the challenging aspects that so far restrained the large scale adoption of such systems by the intended audience: Wearability, real-time operation, pervasiveness, usability, cost. This article is set to present the artificial vision based component of the SoV SSD that performs the scene reconstruction and segmentation in outdoor environments. In contrast with the indoor use case, where the system acquires depth input from a structured light camera, in outdoors SoV relies on stereo vision to detect the elements of interest and provide an audio and/or haptic representation of the environment to the user. Our stereo-based method is designed to work with wearable acquisition devices and still provide a real-time, reliable description of the scene in the context of unreliable depth input from the stereo correspondence and of the complex 6 DOF motion of the head-worn camera. We quantitatively evaluate our approach on a custom benchmarking dataset acquired with SoV cameras and provide the highlights of the usability evaluation with visually impaired users.

## 1. Introduction

Scene understanding is an important topic in a large variety of computer vision based applications, from autonomous driving to assistive systems. Humans generally rely on their visual sense to perform this task. Mobile robots, autonomous cars and visually impaired persons require complex systems and processes based on artificial vision to infer information about the surrounding environment in order to accomplish their tasks or goals that involve mobility and perception.

This paper discusses the design and development of a computer vision based system aimed at assisting the visually impaired persons in accomplishing scene understanding and safe navigation in outdoor environments. The solution presented in this paper is integrated in a sensory substitution device (SSD), Sound of Vision (SoV) [1], a wearable assistive equipment providing acoustic and tactile descriptions of both indoor and outdoor environments. In this context, the actual scene understanding is performed by the user, based on the audio and haptic feedback provided by the SoV system regarding the objects in the scene.

Investigating the state of the art on development of assistive systems for visually impaired, we find that many works have been described in the literature [2,3,4,5,6,7,8,9,10,11,12,13,14,15,16,17,18,19,20]. Nonetheless, from the perspective of the end-user there are still some important steps to be considered before noticeable communities of visually impaired users adopt this technology. The reasons for not having such consumer grade systems largely available for the end-user are mostly related to the incomplete fulfillment of key requirements, out of which some are also technically conflicting: Wearability, real time operation, pervasiveness, functioning in any kind of environments, both indoor and outdoor usability (e.g., offering rich and natural feedback, efficient training instruments, customization possibilities), cost.

The Sound of Vision project aimed to tackle the sensory substitution for the visually impaired in a comprehensive way. That is, it considers all the above challenging aspects and delivers a user friendly, wearable SSD, that works in both indoor and outdoor environments, invariant to the illumination conditions. Real time operation and wearability are conflicting requirements, especially as such systems need to employ complex 3D processing algorithms for environment elements of interest identification. Wearability induces hard constraints on the form factor of the acquisition/processing/rendering devices that can be integrated in the SSD. It also has a significant impact on aspects such as power consumption, heat dissipation.

A minority of the reported assistive systems consider the pervasiveness aspect [18,19,21]. Most work is either in indoor or outdoor environments. This disadvantage mainly comes from the properties of the integrated 3D sensors. The infrared-based sensors, e.g., Kinect, are not meant to handle scenes with bright illumination from the sun. On the other hand, stereo sensors give unreliable depth maps in indoor environments with poor artificial lighting or uniformly colored/textured surfaces. Moreover, the computation pipelines are designed based on the exploitation of reliable depth input coming from high quality sensors, Kinect-like cameras [4,15,22,23] or the Bumblebee stereo camera [3,5,6,14]. With such devices, the wearability constraint is far from being fulfilled.

The systems reported as being reliable in both indoor and outdoor environments deal with different limitations. CASBlip proposed by [2] has an operational range up to 5 m and uses reflected laser light beams for static object detection. The system deals with some problems when used in open environments (outdoor) due to external noises as the sound feedback is not perceived as clearly as it is in indoor environments (e.g., laboratory). [7]’s system relies on a pair of glasses with an embedded stereo camera mounted for depth sensing. Their algorithm is based on ground-plane detection in the disparity domain. Once the ground-plane is computed, the points that do not lie on this surface are marked as obstacles. The drawback of the system is that the accuracy of obstacle detection directly depends on the quality of the disparity maps. In other words, there are circumstance when disparity maps are very noisy and the system fails (e.g., when facing completely uniform areas). Another stereo-based system is the Blavigator, proposed by [14]. It implements a two layer obstacle detection algorithm based on disparity images. One layer contains information located at 2 m in front of the user and it is used for obstacle detection. The other layer, 1 m in front of the user, is used for backup and trajectory correction. The images are acquired with a stereo Bumblebee camera and information is feedback-ed to the user via microvibrators that signals corrections corresponding to the five directions: left, left-diagonally, straight, right diagonally and right.

Some solutions [13,19] use a chest mounted smartphone. In [19]’s case this imposes some limitations on the camera orientation and also on the complexity of the considered algorithms. The major limitation of [13] is the lack of alerting functionalities. Drishti system [21] uses a combination of ultrasound positioning service for indoor navigation and DGPS for outdoor environments. The system cannot accurately predict a person’s height, used for computing the location, as there are only two beacons attached to the user’s shoulder. In this case, the system fails dramatically if the user sits or lies down. Another issue is that the signal emitted from the ultrasound positioning system may be reflected or blocked by furniture or walls resulting in “dead spots”.

While other works like [10,11] yield good processing rates, they lack the usability testing with visually impaired persons. Furthermore, complex machine learning approaches to scene understanding have been reported [24,25,26]. While some of them have been reported to work in real time, either they require processing units not suitable for a wearable system [24], or they cannot infer the information required by an SSD [25]. The latter is the case of single color camera based processing, which, in the absence of structure from motion, cannot provide valuable information (object properties such as position in 3D space and size) for the audio and/or haptic encoding of the scene by an SSD.

## 2. The Sound of Vision System

The SoV system aims to improve both perception and mobility in unknown environments for users with visual impairment. Moreover, it provides feedback to the user in both indoor and outdoor environments and irrespective of the illumination conditions. The extended categories of information that can be provided by the proposed system and its user-centric hw & sw design surpass the limitations discussed in the previous section.

The SOV device aims at improving the lifestyle of visually impaired persons in categories 3 to 5 according to the World Health Organization (http://apps.who.int/classifications/icd10/browse/2015/en). In these categories are included visually impaired persons who require assistance in their daily lives by using a white cane, a guide dog or relying on a guiding person.

Sound of Vision was designed, developed and tested with the strong involvement of end-users, orientation and mobility instructors and specialists in psycho-physics and behavioral science. This multi- and inter-disciplinary approach produced an original hardware and software system, accompanied by a set of innovative and efficient training instruments [27]. The SoV device is foreseen to be used together with the white cane. Still, evidence from the performed usability experiments suggests that, after some amount of training, the users feel confident enough to use it without the cane. Thus, the SoV device provides the user with both redundant and complementary information to the white cane.

The system architecture is based on four repetitive steps: (i) 3D data acquisition from the environment; (ii) 3D environment reconstruction and segmentation; (iii) haptic and audio modeling of the processed 3D scene; (iv) feedback the haptic and audio information to the user.

**Figure 1:**
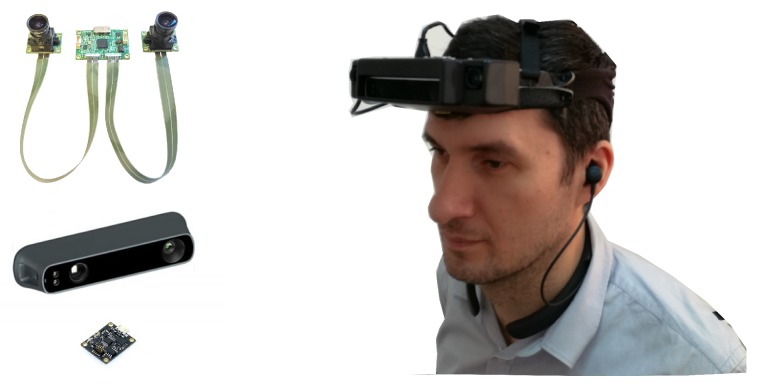
The Sound of Vision headgear: (**left**) 3D acquisition devices integrated in the SoV system—stereo camera (LI-OV580 from Leopard Imaging), IR depth sensor (Structure Sensor PS1080 from Occipital), IMU device (LPMS-CURS2 from Lp-Research) and a prototype implementation of the SoV headgear (**right**).

A general overview of the entire 3D processing component of the Sound of Vision system was presented in [28], focusing on the philosophy behind the design that meets the requirements stated in the previous section. In order to work in both indoor and outdoor environments, and invariant to the illumination conditions, the 3D acquisition system in the SoV device integrates two different types of depth sensors: an IR-based depth sensor (Structure Sensor PS1080 from Occipital, Inc., San Francisco, CA, USA) and a stereo vision camera (LI-OV580 from Leopard Imaging Inc., Fremont, CA, USA). An IMU device (LPMS-CURS2 from Lp-Research Inc., Tokyo, Japan) is also used for recovering the orientation of the head/cameras, as the acquisition devices are rigidly mounted on a headgear (Figure 1). companies, please confirm

The system can be set to work on four 3D acquisition modes: (1) Stereo camera input; (2) structure sensor input; (3) stereo-structure dual input and (4) recording playback. The 3D processing module makes use of different combinations of sensor data to maximize the system usability in different situations and still provide environmental information in conditions atypical to standalone sensors [29]. The fusion of data in the dual mode allows the processing of both color and depth information to detect various structures such as doors, signs, text in indoor environments.

Irrespective of the input, the core approach for scene segmentation relies on efficient detection of the ground (and ceiling, in indoor environments) surfaces, and partitioning of the remaining 3D information in obstacles. Still, different 3D processing approaches are employed to deal with the specific 3D input, structure and composition of indoor and outdoor environments, respectively. The most significant elements required in navigation scenarios are related to avoidance of obstacles and dangerous situations. In such scenarios, the user experiences a rapid change of scene structure, and thus a frequent change of elements encoded by the system. The aim is to keep the number and type of elements signaled by the system low enough, such that the user is able to understand the scene while moving.

Two main scene encoding models were designed and implemented in the system: a continuous one and an iterative scanning [30,31,32]. The former provides the user with continuously updated feedback based on encoding a labeled depth map of the scene. The latter works by iteratively scanning the environment with an expanding sphere and producing specific sound/haptic patterns for the objects as they are intersected by the sphere. In any case, the user feedback is also augmented with information regarding dangerous situations, such as the presence of negative obstacles (e.g., holes in the ground, stairs going down) or elevated (e.g., head-height) objects on the path.

While the continuous mode mainly relies on the detection of the presence/absence of obstacles and removal of the ground surface (which is not encoded), the scanning mode requires precise information regarding the objects in the scene (e.g., position in 3D space, width, height, type) to produce the patterns.

The output is rendered to the user by means of hear-through headphones and/or a custom designed haptic belt. The type of patterns (sounds and/or vibrations) and the selection of several other modifiers of the encoding (e.g., direction of the best navigable space) is highly and easily customizable by the user.

The main novelties brought by the Sound of Vision compared to state of the art devices performing sensory substitution for the visually impaired are:

- It helps VIPs both to perceive the environment and to move independently, without the need for predefined tags/sensors located in the surroundings;
- pervasiveness—the system works in any kind of environment (indoors and outdoors) and irrespective of the illumination conditions;
- it provides rich and naturalistic descriptions of the environment through original combinations of audio and haptic encodings;
- it provides a real-time description of the environment to the users, i.e., the description is continuously updated, fast enough such that the users are able to perceive the environment even when walking;
- it is accompanied by efficient training programs and tools, based on modern techniques (virtual and augmented reality, serious games), developed together with Orientation & Mobility instructors and specialists in behavioral science.

In this paper, we detail the core outdoor processing pipeline integrated in the 3D component of the SoV system. The proposed solution relies on an incremental reconstruction of the scene from consecutive frames provided by a stereo camera. We introduce a confidence-based estimation of the 3D information as well as a color-based refinement in order to cope with the inherent errors and uncertainty of the stereo-based measurements. Furthermore, we develop a novel approach to efficiently cope with the dynamics of scene elements under the constraints of the incremental reconstruction. To provide real-time feedback, we rely on fast 2D based segmentation methods and exploit parallel computing on the GPU.

## 3. Stereo Based Reconstruction and Segmentation in Outdoor Environments

### 3.1. Overview of the 3D Processing Pipeline

**Figure 2:**
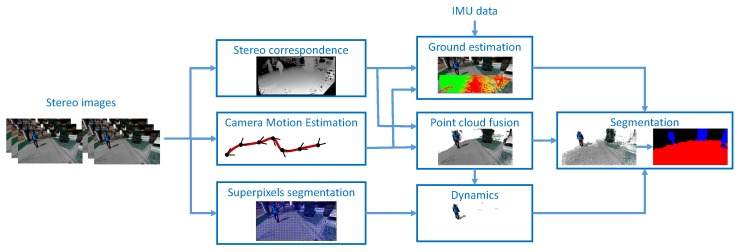
Overview of the computation steps in our stereo-based reconstruction and segmentation system.

The outdoor processing component (Figure 2) employs a 3D reconstruction method that generates a global 3D model. This model is consistent along the timeline of the system use. Stereo stream processing while incrementally adding the 3D representations of individual frames increases the consistency of the 3D model. This global 3D model is always represented in the camera coordinate system of the current frame.

The global 3D model mainly consists in a 3D point cloud, which is further segmented into objects by processing various histogram representations of the disparity maps. The elements of the environment are tracked between frames based on tracking the labels assigned to the corresponding 3D points in each frame. This allows their temporal identification to be consistent for the entire stream.

**Figure 3:**
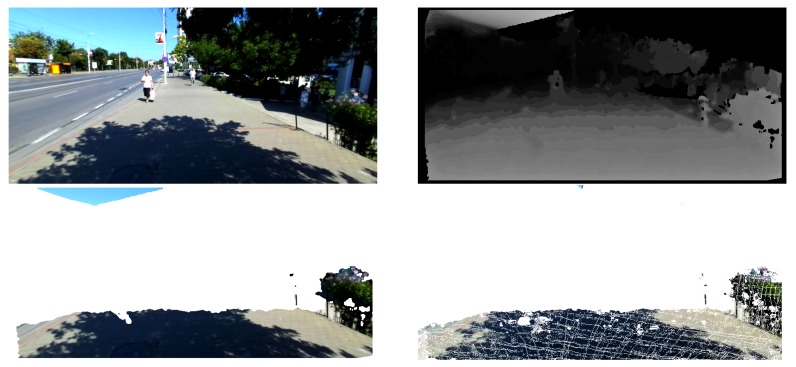
Comparison between our reconstruction and single frame based reconstruction. (**Top-left**) RGB image from the left camera. (**Top-right**) Disparity map computed with the approach of [33]. (**Bottom-left**) 3D reconstruction constrained within 5 m in front of the camera, based solely on the disparity map of the current frame. Errors in the estimation of the stereo correspondence lead to the presence of false structures in the reconstruction. (**Bottom-right**) Result of our incremental reconstruction showing the accumulated stable points (confidence above a predefined threshold).

The reconstruction of a global 3D model is fundamental because it can be the solution in managing the high amount of errors in the computation of depth from disparity. If each frame is independently segmented in the presence of these errors, then the detected objects might have large errors and thus the system becomes unreliable as shown in Figure 3. By introducing a confidence evaluation for the 3D measurements we are able to avoid false positive structures in the reconstruction. Moreover, intermittent lack of disparity measurements can also be compensated with 3D information reliably reconstructed in the previous frames. The confidence of a 3D measurement is computed based on the number of frames in which it could be tracked, as detailed in the following section.

A specific issue with such an incremental approach to the reconstruction is represented by the inherent exclusion of dynamic structures from the confident set. In order to solve this problem we propose to exploit the color information in the acquired images by identifying significant color variations between corresponding depth measurements of consecutive frames. In order to efficiently extend these specific points to the larger dynamic structures to which they correspond we use a generic over-segmentation method in the color space (superpixels segmentation) and a statistical approach.

The current validated point cloud (composed of confident 3D measurements and dynamics) is then segmented into structures of interest: ground surface and positive and negative obstacles. Other elements of interest like doors, signs, texts are detected based on color information only and are not part of the core pipeline detailed in this paper. We employ a fast segmentation method in 2D space by computing various histograms of the 3D point cloud.

Out of the positive obstacles, it is also useful for the mobility of the blind user to distinguish the walls (or other large surfaces perpendicular to the ground, like fences) that can be exploited as a shoreline in navigation. To this end, we employ an heuristic based on the properties of the detected 3D structures (i.e., size, orientation of the best fitted planar surface with respect to the ground) to identify wall-like objects.

Passing between two obstacles requires the user to mentally calculate the navigable space between them based on their properties provided by the SoV system (i.e., widths and 3D positions of the centers). In order to aid the navigability between obstacles in cluttered environments, the system also provides information regarding the best navigable direction. This information is computed in the last stage of the 3D pipeline based on a histogram of radial directions of the measurements corresponding to the detected positive and negative obstacles.

### 3.2. 3D Reconstruction

The system relies on passive stereo vision, so, in order to perform the incremental reconstruction we need to employ three operations for each frame: (1) Estimation of depth data from stereo; (2) estimation of camera motion with respect to the previous frame; (3) fusion of current frame data into the reconstruction. In the following we detail these parts of the reconstruction process.

**(1) Estimation of depth:** In order to work properly, the stereo vision system requires a set of intrinsic and extrinsic parameters which can be used to obtain a correct rectification of the acquired left and right image. These parameters are computed in an off-line calibration process, while rectification, which ensures distortion removal and stereo alignment, is an on-line process. Once these two processes are completed, the most important task is solving the correspondence problem between the left and right image. We use the stereo correspondence method by Geiger [33] to compute a dense disparity map. We employ a parallelization of the original algorithm in order to produce a dense disparity map, without the need for a global optimization, and still stay within the real-time operation constraints. The depth values, $z_{ij}$, are computed based on the disparity values using the equation $z_{ij}=bf/d_{ij}$, where $d_{ij}$ is the disparity of pixel (i,j) in the image, *b* is the stereo baseline and *f* is the focal length of the camera.

**(2) Camera motion estimation:** In order to perform the registration of the previous measurements with the camera coordinate system of the current frame, we need to estimate the camera pose in the current frame. This is a complex task generated by a combination of the motion of the head mounted stereo camera and body motion. The recovery of this 6-DOF motion needs to comply with the high update rate required by the application. The performance evaluation of any camera motion estimation technique needs to include: high accuracy in relation with ground truth, real-time behavior, robustness to motion-blur induced by body motion. In the end we selected the camera motion estimation technique proposed by Geiger in the *libviso2* framework [34]. This technique is frequently used in automotive and robotics applications. For applications with head-mounted cameras, the internal parameters required adaptation. For parameter tuning, a 3D virtual environment was designed. This allowed the generation of benchmark testing stereo sequences for human assistive devices in different outdoor illumination conditions and recreate some special situations of real life environments. The tests revealed valuable data to be processed for computing the efficiency of the algorithms while acknowledging the worst case scenarios. In addition, tests revealed the highest attitude errors to be equal to ±10 degrees for orientation and ±0.1 m for position. These errors establish the confidence interval that maintain the system reliable, as showed in Section 4. Based on the collected data, the internal parameters of the libviso framework were tuned for the best performance of our stereo camera’s motion estimation.

In the current design the IMU is used to improve ground detection. Future refinements will include the visual-inertial fusion for motion estimation.

**(3) Point cloud fusion:** Most point-based 3D reconstruction methods map all valid pixels to 3D and project them into a common coordinate system according to the estimated camera pose. Nonetheless, storage requirements can grow rapidly if the association problem is not solved. Furthermore, redundant information cannot be used for improving reconstruction accuracy. On the other hand, classic multi-view optimizations such as bundle adjustment are computationally unfeasible for dense real-time reconstructions such as the proposed one.

Instead, we use an approach for solving the association problem based on the re-projection of the global 3D model onto the image plane of the new frame. Merging is performed based on both color and depth similarity. The coordinates of the resulting point are obtained by computing the 3D mean of the fused points. This approach reduces the number of points which have to be stored, and also leads to higher accuracy by averaging out measurement noise over several frames.

Since the reconstructed sequences can be very long, the size of the global 3D point cloud grows rapidly. In order to prevent excessive memory requirements and high computation times associated with processing a large number of points in the point cloud, we devise a ‘soft reset’ of the global model. This is achieved by only keeping track of the global 3D model that intersects a certain volume of interest (VOI) defined in the 3D space around the user. The VOI is defined in the form of a user customizable bounding box with coordinates [left, right; up, down; front, back] with respect to the user (camera) position. This approach also ensures a proper filtering based on proximity for the objects signaled by the system in order to avoid overwhelming the user with excessive information.

Each 3D point that belongs to the global model is linked to a confidence measure. This measure is linearly dependent to the number of frames in which the points could be tracked. Tracking a point is equivalent with its presence in the sensor’s field of view and the disparity computation algorithm being able to generate a 3D measurement for it.

In each frame, the global 3D model is transformed in the camera coordinate system of the current frame and is updated with new data by:

1. Updating existing points based on new measurements, in case they can be fused, i.e., their 3D measurements match within a predefined distance threshold; with each new update, the confidence of the 3D point is increased; these points are recorded in a 2D fusion map;
2. Adding new points which have not been previously measured by the sensor;
3. Removing garbage points, which either remain out of a volume of interest around the camera, or did not gain enough confidence since they have been first measured.

The further segmentation of the global model is performed only considering the 3D measurements with a confidence above a minimum threshold. This allows the removal of possible outliers. The outliers can emerge not only due to disparity estimation inaccuracies. The camera motion estimation is based on an optimization process and thus, it can also introduce errors in the reconstruction process. These errors are specific to fast rotations of the camera, when the motion blur considerably affects the tracking of points between frames.

**Reconstruction of dynamic objects:** While introducing the confidence measure for the 3D points increases the accuracy of estimating static regions in the environment, it also intrinsically removes the dynamic objects. In such circumstances, the estimation of dynamic regions is a very challenging task as they are represented by 3D points that cannot be fused with existing ones in the global model due to their movement. While the dynamic regions in consecutive frames can partly overlap, their corresponding 3D points cannot sufficiently grow in confidence and would not be included in the segmentation process.

State of the art solutions for estimation of dynamics either rely on accurate depth data acquired with time-of-flight sensors [35], or consider the dynamics estimation problem only for removing such regions from the reconstruction [36]. To cope with the contradicting requirements, we exploit the color consistency between consecutive frames. Commonly, frame alignment is a result of image stabilization, a process equivalent to applying a 2D homography transform. However, an approach based on dense optical flow requires a high amount of computation resources to achieve the appropriate accuracy. We use the already available remapping of 2D points of frame *t* into points in frame *t + 1* to avoid such calculations. The 2D points at time *t* are computed based on the re-projection of the motion-compensated 3D points forming the global model on a synthetic depth map. Using this estimation of the optical flow from depth measurements, we can remap the color images allowing for point-wise comparison. The resulting color difference map also accounts for the 3D measurements unavailable in the current frame and for new 3D measurements in the current frame with no corresponding measurement in the previous ones.

Using both the fusion map and the color difference map, a new measurement is considered dynamic based on the following constraints:

1. The new 3D point could not be fused with an existing one because no measurement was previously performed for that position OR
2. The fusion of the new 3D point with and existed one, marked as dynamic in the previous frame, was attempted but could not be performed OR
3. The fusion of the new 3D point with an existing one, marked as static in the previous frame, was attempted but could not be performed AND the color difference map in that position contains a value larger than a threshold.

**Figure 4:**
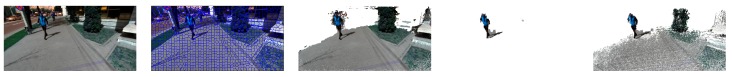
3D reconstruction with outlier filtering in the presence of dynamic elements. From **left** to **right**: Image from the left camera; superpixels segmentation; accumulated point cloud; superpixels marked as dynamic; point cloud of confident and dynamic points. artifacts from the moving person and outliers from stereo correspondence and camera motion estimation are filtered out as they are not tracked over a minimum number of frames.

The last constraint ensures that non-fused 3D points are considered dynamic only if they also sufficiently differ in color. Thus we dramatically reduce the effect of depth computation errors in the estimation of dynamics. This effect is further reduced by employing a segmentation of the dynamic regions in the color image. The segmentation method is based on a statistical approach and aims to refine the dynamics estimation. It corrects the 3D points representing false negative and false positive detections. The proposed method is based on superpixels segmentation of the color image. Then, the superpixels are marked as static or dynamic based on the statistics of their corresponding 3D points that are found to belong to dynamic objects (see Figure 4). The superpixel segmentation of the image is performed on the GPU and is also used for refining the following object segmentation steps. Using the superpixel segmentation we avoid the expensive region growing technique suggested by [35] for dynamics segmentation.

To correct for the false positive and false negative dynamic labeling, all the 3D points corresponding to dynamic superpixels are marked as dynamic, while all the points corresponding to static superpixels are marked as static, irrespective of their initial labeling.

In each frame, the dynamic 3D points that are not fused with new data are removed from the global model. This ensures the elimination of artifacts from the moving objects. Still, this approach allows the dynamic points to gain confidence when a dynamic object becomes static.

### 3.3. Segmentation

**Ground processing:** The detection of objects in the environment requires a prequel step consisting in ground surface computation. The ground surface is detected by first estimating the equation of a plane that approximates this surface based on a row-histogram of the current frame’s disparity map. We build on a previously developed method [37]. In the second step, all the 3D points in the global model that fit this plane equation within a threshold are considered part of the ground surface. The value of the threshold was empirically chosen with a value of 15 cm, which satisfies both the usual slight unevenness of the real ground surfaces and for 3D point estimation errors. Furthermore, uneven surfaces within this oscillation of level generally do not pose threats to the safety of VIP’s mobility, especially when also using the white cane. To increase the reliability of the estimation, the detected ground plane is tracked across consecutive frames using a cross validation against the estimated camera motion and a maximum allowed variation of the camera height above ground between consecutive frames.

**Figure 5:**
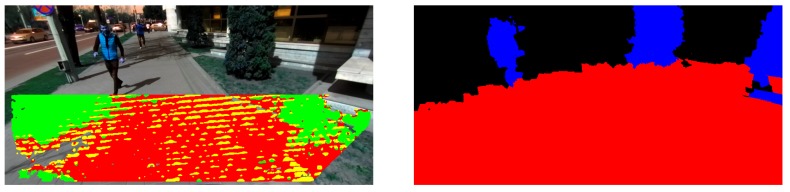
Example segmentation result. (**Left**) Initial ground surface detected using the approach based on the row histogram of the disparity map. Points marked with red fit the estimated ground plane within a 15 cm threshold. Points in yellow fit this plane within 20 cm error and the ones in green are below this plane at more than 20 cm. (**Right**) Labeled superpixels as a result of ground refinement and obstacle segmentation.

This approach manages to identify a large part of the 3D points that lie on the ground surface. Still, any points incorrectly marked as non-ground can lead to false positive obstacle detections. In order to keep the computation low, the fitting is performed against a planar structure even though it is not the optimal representation of the ground surface [38]. In order to overcome this limitation and avoid an expensive fitting of a higher degree surface, we employ a fast statistical approach for growing this initial segmentation, similar to the solution for estimation of dynamic regions. Thus, we exploit the superpixels segmentation and first mark the ground superpixels based on the statistics of their corresponding ground points found with the planar fitting in the current frame and those already marked as ground in the previous frame. Next, all the other 3D points projected on superpixels labeled as ground are also marked as ground (Figure 5).

The ground processing component is also responsible with the detection of regions corresponding to negative obstacles. These can be represented by holes in the ground, potholes or any large difference of height between two ground surfaces (e.g., edge of a railway station platform, stairs down). The system detects their presence based on empirical assumptions regarding their 3D characteristics [39]. The reliability of this approach is ensured by employing a tracking mechanism in order to validate the identified candidates.

**Obstacle detection:** The point cloud segmentation is performed in the 2D space as it is less computationally demanding than 3D approaches. The principle of this approach consists in building and processing the U-Disparity histogram for the global 3D point cloud, excluding the points corresponding to the ground surface. The histogram is constructed by only considering the 3D points marked as stable (having a confidence above the threshold) and the ones marked as dynamic. The computed U-Disparity map represents a column-histogram of the disparity map obtained by 2D re-projection of the current point cloud.

The computed histogram is processed with morphological dilation and erosion. This ensures both the removal of small outlier regions and filling missing regions of larger object surfaces. Moreover, regions corresponding to objects very close to each other (with respect to horizontal, vertical and depth alignment) are merged in a single region. This form of under-segmentation ensures that the user perceives a large space occupied with multiple objects as a single obstacle. This way we avoid the sensory overload induced by providing information on several objects instead of a merged obstacle. When the user approaches such spaces, the separation of objects is also translated into a separation of the corresponding regions in the histogram, thus ensuring a detailed description of the objects in the close proximity. The actual segmentation is performed using a connected components approach.

Each region in the segmented U-Disparity map is assigned on object identifier which is also transferred to the corresponding 3D points of the global model. The object regions are then identified in the 2D image by re-projecting the identifiers of the 3D points. These regions are further refined using the superpixels segmentation. Each 3D point is assigned an object identifier based on an identifier computed for its corresponding superpixel. The superpixel identifier is computed based on the dominant identifier projected on its surface.

**Best free space:** Providing the user with information about the most favorable navigable space in front is very practical in mobility scenarios. This information specifies the direction (azimuth) the user can maneuver and the depth of the free space in that direction (in meters). In case of multiple detection, the slice closest to the camera heading direction is chosen to be the best free space. Moreover, a minimum width of free space is considered (0.9 m), equivalent to a standard door opening. The slices of navigable space are computed based on radially sampling the detected obstacles (hanging, on the ground and negative ones).

The free space is detected in a θ-Disparity map computed from the current 3D reconstruction [40]. The significant structures in the scene are radially represented in relation to a point of interest, by applying over the disparity values of the point cloud a specific polar transformation. The pole of the transformation is the middle pixel of the bottom line of the reprojected disparity map. This corresponds to the position of the camera. The new map shows the set of disparity values that are laying along the direction angle θ in the original disparity map, D(x,y), relative to the pole of the transform. The θ-Disparity map is obtained by computing a column wise histogram of this polar map. A weighting factor sin(θ) is used on each element in order to emphasize the nearby obstacles and to avoid the noticeable degeneration in the polar transformed disparity map. In the vicinity of the extreme angles (equal to 0 and 180), the values of the pixels corresponding to those columns tend to be similar.

The θ-Disparity map has only positive integer values and each point indicates the number of points from the point cloud that lie across a certain direction and have a particular disparity value *d*.

## 4. Implementation and Performance Evaluation

### 4.1. Technical Performance

The evaluation of the SoV prototype was performed using a mobile computing unit with an Intel(R) Core(TM) i7-7700HQ CPU @ 2.80 GHz, 8.00 GB RAM; GPU: nVidia GeForce 960 m and works at 7 fps in stereo vision mode. In the context of our application, it seems that the update rate is sufficient to allow the blind user to perceive the environment even while moving (as concluded from the mobility tests described in the next subsection). We heavily exploit multi-threaded execution on the CPU and GPU to keep all other required computations within a low frame rate when the dense disparity computation alone runs at maximum 10 fps on the SoV computing unit. Still, stereo correspondence, camera motion estimation and superpixels segmentation are independent steps that can be run in parallel. We believe that the announced advent of improved stereo vision hardware, providing both small form factor and weight and speed of hardware-based dense disparity computation, will allow us to further alleviate the computational burden for the SoV device and substantially increase its performance or power consumption, as required. For segmentation we use a GPU implementation of the SLIC superpixels [41]. The point cloud processing runs on the GPU as well, including the computation of U-, V- and θ-histograms.

The headgear mount of the stereo cameras allowed us for an approximately 15 cm baseline. We used the resolution of the stereo images of 2 × 1280 × 720 pixels @15 fps. For the incremental reconstruction, we impose a confidence threshold of 5 frames for the static points and perform the fusion of old and new 3D measurements within a distance of 20 cm. The fusion distance was derived based on the intrinsic and extrinsic parameters of the stereo camera used which induce a limitation to a 0.26 m accuracy for the depth estimation at a distance of 5 m from the camera. The ‘soft reset’ is employed when the confidence of a 3D point doesn’t reach the threshold in maximum 10 frames. When refining the segmentation of ground and dynamic objects, we mark a superpixel as ground/dynamic when 50% of its corresponding 3D measurements are labelled as ground/dynamic. We impose a maximum number of 500 superpixels per frame.

**Figure 6:**
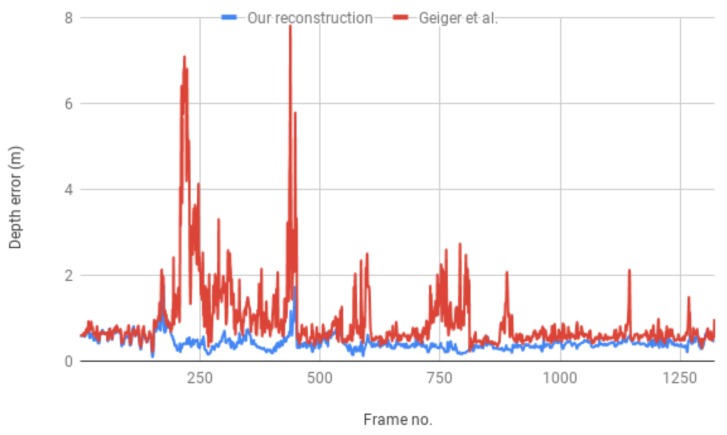
Average depth error of the reconstruction compared to the depth data from the structured light camera.

In order to evaluate the accuracy of our reconstruction we use the depth measurements from the structured light depth camera mounted on the Sound of Vision headgear. For evaluation we use data captured by the system running in dual mode under ideal illumination conditions for the IR camera (e.g., cloudy weather or evening light to avoid interference with the IR radiation from the sun). In dual mode, the system acquires frames from both devices, the stereo and the depth camera, synchronizes and registers them based on a previous calibration of the ensemble [29]. We use the depth measurements from the structured light camera as ground truth and compute the reconstruction error for our method, i.e., the distance (in terms of depth) from the ground truth after re-projection to the image plane. We evaluate the reconstruction in 1321 images of static and dynamic urban environments. The results are summarized in Figure 6. We measure a 9% average deviation of our incremental reconstruction from the ground truth, compared to a 17% deviation computed for the single frame reconstruction based on Geiger’s approach [33]. This corresponds to an overall improvement in reconstruction accuracy of almost 10% over the initial depth estimation. However, for sequences in which there are many moving objects, we obtain an improvement of only 5%, as in this case, considerable parts of the reconstruction correspond to dynamic regions for which our method takes the measurements directly from the disparity map.

For accuracy benchmarking purposes we prepared a dataset consisting in 111 manually annotated frames acquired with the SoV stereo camera. The images were recorded outdoors, in various scenarios, including controlled scenes (setup with walls and cardboard boxes used in our usability experiments) and operational ones (uncontrolled urban scenes, e.g., park, sidewalk, parking lot). For a subset of the images the annotations were performed for both a 5 and a 10 m extent of the environment in front of the camera. This allowed us to evaluate the results of our system for the two most common configurations suggested by our users.

The accuracy of obstacle detection was evaluated using two approaches. On one hand, a pixel-wise comparison between the ground truth segmentation and the results of the device was employed in an automatic evaluation framework developed within the project. In this framework, a detected element is defined as a true positive based on the Jaccard similarity coefficient (Intersection over Union) between its bounding box in the image and the bounding box of the corresponding annotated (ground truth) region.

**Figure 7:**
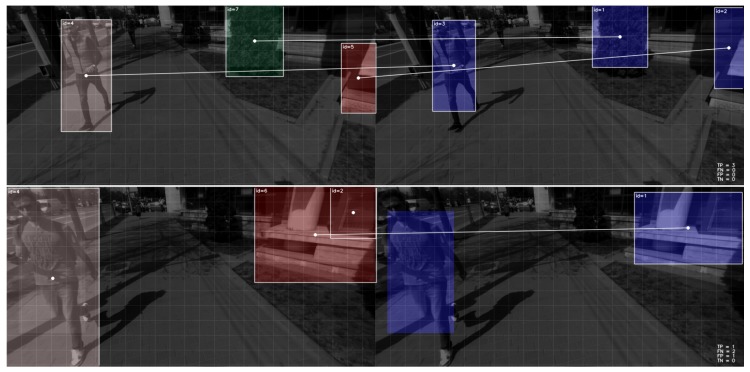
Examples of automatically established correspondences between detected elements (**right**) and ground truth (**left**) for evaluation purposes. Top image shows correct identification of correspondences based on a 0.5 value for the similarity coefficient. The bottom image is an example of a frame for which a manual refinement of the correspondences was performed to account for the missing correspondence in the case of the pedestrian and to account for the clustering of the building elements into a single object.

The ground surface is detected with 100% correspondence between our results and the ground truth and an 0.92 average similarity coefficient over all evaluated frames. Obstacle detection results are less relevant with this approach. The automatic evaluation process only supports one-to-one matches (no splits and merges) and marks a correspondence between the detected element and a ground truth one if the similarity coefficient of their bounding boxes is larger than 0.5. This leads to improper correspondences between ground truth and the results of the segmentation (Figure 7). Thus, to evaluate the obstacle detection results we manually refined the correspondences reported by the evaluation application to correct the false and negative correspondences and account for clusters of objects in the environment that we purposely and by design report as single objects. We obtain a 0.93 precision, with 0.90 recall and 0.85 accuracy. At the risk of introducing a level of subjectivity in the evaluation, we believe this approach better reflects the evaluation results against the expected output, i.e., it is a more user-centric evaluation of the results.

On the other hand, another such user centric evaluation of the detection results was also performed, in which the presence/absence of specific and generic objects was accounted for. This evaluation is less expensive to run as it does not require time-consuming manual annotations of ground truth and allows for an extensive assessment over a larger and more diverse set. For example, the detection of the ground plane was evaluated against 10,475 frames, resulting in 2.1% false positive detections, and 3.4% false negatives. Detection of negative obstacles (missing sewer caps, holes in the ground, railway platform, stairs down) was evaluated against 924 instances in 790 frames resulting in 0.87 precision and 0.90 recall. The assessment was performed on a dataset of images recorded with the SoV system by sighted and visually impaired persons in operational environments.

Overall, we noticed similar results obtained for both extents, 5 and 10 m, in terms of presence/absence of obstacles with a higher degree of under-segmentation of further away objects. A higher number of objects are usually present as the extent of the VOI is enlarged. Rendering all of them to the user, can easily lead to a sensory overload. We believe that extending the encoded space to distances larger than 5 m can be useful in large empty spaces, where visually impaired people could, in general, easily loose the sense of orientation.

### 4.2. Usability Assessment

The development of the Sound of Vision system was approached in an iterative manner, involving three prototypes. Each version of the system was built based on the results of technical and usability experiments with the previous one. The first two rounds of usability testing were performed in custom virtual environments and laboratory setups (predefined scenes with cardboard boxes). The results helped us evaluate and improve the usability of the audio and haptic encodings. Furthermore, they also provided valuable insights for improving parts of the 3D processing pipeline. A relevant example is the computation of objects properties and its impact on user perception [28]. All the experiments were designed with increasing difficulty and were interspersed with training. In the last round of testing, we introduced the operational environments (real life, indoor and outdoor scenarios). An extensive report on the results of these usability experiments is not in the scope of this paper. Still, we would like to provide some highlights of the results obtained in outdoor environments with the stereo-based pipeline.

**Figure 8:**
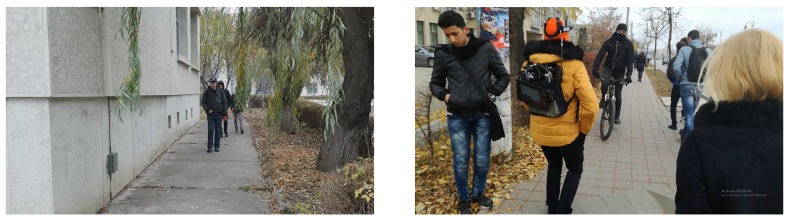
Examples of outdoor testing scenarios: (**left**) Semi-controlled environment, in which the test consisted in walking along a building wall while avoiding hanging and ground-level obstacles; (**right**) uncontrolled environment, in which the test consisting in walking on a crowded sidewalk.

The main research questions addressed aspects regarding the usability in real life environments and under real life circumstances (outside laboratory setups): Whether the visually impaired users are able to identify obstacles and specific objects (negative obstacles, hanging obstacles, signs, walls) which define the added value of SoV compared to using the white cane; whether they are able to move around, avoid obstacles, identify targets (e.g., bus stop, corner of a building); how is their mobility performance with the SoV system compared to traditional assistive devices (i.e., white cane). The outdoor tests run at the Technical University of Iasi involved four visually impaired users (categories 4 and 5 of blindness) who went through two categories of experiments: perception (ego-static scenarios) and mobility (ego-dynamic) tests. In the mobility tests, two types of scenarios were considered: semi-controlled environments and uncontrolled environments (Figure 8). The VI users were asked to walk on predefined routes fulfilling specific tasks. By semi-controlled environments we denote natural areas, usually containing short testing routes (15–30 m long), with no or light traffic, for which the testing team could control the structure of the scene. This ensured the presentation of the same scene and tasks to all VI participants. Specific static and/or dynamic obstacles (i.e., people) were purposely and systematically introduced in some testing scenarios. By uncontrolled environments we understand public areas, with varying, uncontrollable traffic. This only allows for qualitative (as opposed to quantitative) performance evaluations and comparison of performance between users and modalities (i.e., white cane, SoV + white cane, SoV). Still, it better reflects the usability of the system in operational environments.

With minimal training in using the SoV system outdoors, both perception and mobility in the environments were achieved with very good accuracy. The experiments revealed a very high effectiveness (i.e., task completion rate) of the SoV system with very little training: considering that a task was successfully completed if the accuracy per task was above 85%, the overall performance analysis revealed an effectiveness of 88.5% for the SoV device, considering all four users in a number of 65 experimental scenarios. This proves both the intuitiveness in using our system and the efficiency of the devised training programs and tools. The Sound of Vision project gave an exceptional attention to training. Besides the general limitations of the visual rehabilitation itself, the lack of efficient training programs and tools is considered to be an important factor that hinders the adoption of such SSDs by large communities of visually impaired [42,43]. Thus, the project devised efficient training resources, relying on valuable contribution from neuroscience, psychophysics and behavioral science specialists, best practices provided by Orientation and & Mobility instructors and on modern concepts like serious games. The above results of the usability assessment were obtained by the visually impaired users after three two-hour sessions of incremental training with the SoV system in virtual environments and indoor environments, followed by another four two-hour sessions which included training and testing in outdoor scenarios.

When comparing SoV with the white cane, we found out that, for our sample of rather inexperienced white cane users, mobility with the SoV device was accomplished with performance comparable to the performance with the white cane, and sometimes better.

The SoV system offers the clear advantage of informing the user about the presence of objects which could not be otherwise detected with the cane (head level objects or signs) or that could be missed by it (holes in the ground).

Moreover, the system allows a good perception of walls which are very much used by VI people as a shoreline during navigation. With the SoV system, users could walk at a reasonable distance from the wall, without the need to hit it with the cane (average of 23 cane hits when using the white cane only, compared to 3.5 hits when using the white cane and SoV on a 30 m path) and could easily detect the corners of the buildings (with a 0.92 average accuracy over all tests).

For inexperienced white cane users, mobility with the SoV device was accomplished with performance comparable to using the cane, and sometimes better. Users more experienced in using the white cane tend to rely more on the cane than on the system, when provided with both assistive devices. Less skilled white cane users chose to rely more on the SoV system.

The added value of SoV compared to the white cane was reported by the participants to consist in: providing early feedback about static and dynamic objects (at a larger distance than the white cane), providing distinctive feedback about head-height objects, walls, negative obstacles and signs.

A selection of videos showcasing some of the usability experiments can be accessed on the YouTube channel of the Sound of Vision project (https://www.youtube.com/channel/UC5MSSMmEG1ABPZDxzPIZoWQ), one of the most ilustrative for the outdoor evaluation being https://www.youtube.com/watch?v=3XlR2RNy8cg.

## 5. Conclusions

Scene understanding and safe mobility are key aspects in the effort of improving the lifestyle of visually impaired people. Vision restoration through multimodal representations of the environment (sound and haptics) represents one of the most promising solutions for aiding the visually impaired. Sound of Vision is a concept that goes beyond the state of the art of the visual sensory substitution systems having the potential of becoming an affordable commercial device that will actually help blind people. It was designed by giving high importance to the possible limiting factors: Wearability, real time operation, pervasiveness, training, evaluation with end-users. To achieve these challenging requirements, the SoV SSD relies on complex computer vision techniques and a fusion of sensors to provide users with a scene description in any type of environment (indoor, outdoor, irrespective of illumination conditions).

In this paper, we have presented a robust and accurate approach for the reconstruction and segmentation of outdoor environments, based on the data acquired from the SoV stereo camera and IMU. The architecture design for stereo-based reconstruction and segmentation is a major contribution. In addition, ground processing, dynamic obstacles detection and scene segmentation can be considered novelties in the vision pipeline. Due to their original purpose, other algorithms such as disparity estimation and camera motion estimation were tuned for the particular cameras used in our system and for the degrees of freedom induced by the current application. At the core of our approach is a fusion of stereo depth maps in a 3D model (point cloud) that is efficiently represented and processed on the GPU. The system provides a reliable 3D representation of the environment even with noisy depth data and in the presence of dynamic elements. We have demonstrated our system’s performance on a large custom dataset recorded with the SoV cameras. The evaluation performed with end-users indicates that the system could be successfully used for perception and safe mobility in a variety of real-life outdoor scenarios.

The prototype implementation of the SoV system was showcased to work on a processing unit in the form of a laptop. We intend to deploy the whole processing pipeline on a mobile system such as the NVidia systems-on-chip. Preliminary evaluations were performed with the stereo-vision based component of the SoV system running on the TX1 SOC. The results show that we can achieve a 3 fps update rate without any architecture-specific optimizations. The main bottleneck is represented by the time required for computing the disparity map on the ARM CPU. Nevertheless, we believe that the integration of the recent hardware developments regarding mobile computing and stereo vision (e.g., the nVidia AGX Xavier SOC, the ZED mini stereo camera) will allow us to overpass this limitation.

In the future we would also like to add a semantic layer upon our obstacle detection system. Preliminary results with such an approach already indicate that, besides providing the users with more specific information regarding the environment, it can also be used to further improve the segmentation results (by filtering out false positive objects and merging over-segmented regions). Furthermore, we will extend the technical and usability evaluation in outdoor environments to a larger sample and in more diverse conditions.
